# Mutation Detection in the Menkes Gene *ATP*7A Using the Protein Truncation Test

**DOI:** 10.4137/cpath.s565

**Published:** 2008-06-19

**Authors:** Lisbeth Birk Møller, Nina Horn

**Affiliations:** Kennedy Center, Gl. Landevej 7, 2600 Glostrup, Denmark

**Keywords:** protein truncation test, X-linked, menkes disease, ATP7A, mutation, screening

## Abstract

Menkes disease (MD) is a rare recessively inherited lethal disorder of copper metabolism. The gene *ATP7A* defective in MD consists of 23 exons and the coding region encompasses 4500 bp. About 300 distinct mutations, representing all types, have been identified in *ATP7A.* However all mutations identified so far in the exon 2 to exon 7, corresponding to 1869 bp of the coding sequence, result in truncated protein products. No missense mutations have been identified in this region. As about 30% of the total number of mutations identified are located in exon 2 to exon 7, we have designed a protein truncation test (PTT) for rapid detecting of mutations in this part of the gene. In order to determine the applicability of the test, we analysed RNA obtained from eleven MD patients with known mutations in this region. As a truncated product could be identified in all the included samples, PTT proves to be a useful technique for rapid detection of mutations in the N-terminal part of the *ATP*7A gene. Furthermore as MD is a X-linked disease, normally only affecting boys, the risk of false negative results, due to nonsense mediated RNA decay, leading to allelic exclusion, can be left out of account.

## Introduction

The human disorder Menkes disease (MD) is a rare X-linked recessive lethal disorder of copper metabolism (Menkes et al. 1962), which is characterised by mental retardation, severe neurological degeneration and connective tissue abnormalities (for review see, Kodama and Murata, 1999). The disease is caused by mutations in the *ATP7A* gene, which encodes a Cu-transporting P-type ATPase involved in copper efflux from cells and in intracellular transport of copper to copper-requiring enzymes (for review see, Lutsenko et al. 2007). The ATP7A protein is normally localized to the trans-Golgi network, but translocates to the plasma membrane in response to increased copper concentrations. To a large extent the clinical features of Menkes disease can be attributed to malfunction of one or more copper-requiring enzymes, such as lysyl oxidase, cytochrome C oxidase, or dopamine beta hydroxylase, caused by the deficiency of ATP7A (Kodama and Murata, 1999).

ATP7A consist of 23 exons, and the ATG translation initiation codon is located in exon 2. To date, others and we have identified a total of more than 300 different mutations in the ATP7A gene in about 400 unrelated MD patients with the classical severe form or with one of the atypical phenotypes (Tümer et al. 1997; Tümer et al. 2003; Møller et al. 2005; unpublished results). Methods used to identify the mutations include Fluorescent in situ hybridization (FISH), Southern blot analysis, polymerase chain reaction (PCR) amplification of individual exons, Multiplex PCR, Reverse transcription PCR (RT-PCR), Single-strand conformational polymorphism (SSCP) and Dideoxyfingerprinting (DDFP). The mutations, representing all types, are distributed fairly evenly over the entire coding DNA, from exon 2 to exon 23, although the various types of gene mutations are not. Deletions, insertions, nonsense and splice site mutations are found scattered all over the gene, whereas missense mutations tend to be clustered in conserved domains of the ATPase core. To our knowledge, no missense mutations have so far, been observed in the copper-binding region of ATP7A encoded by exon 2 to exon 7. Because all the mutations identified in exon 2 to exon 7, lead to premature termination of the Menkes protein, it was tempting to evaluate the efficiency of the protein truncation test (PTT) for identification of mutations in this region.

PTT is based on cell-free transcription and translation of RT-PCR amplified target mRNA. Proteins of lower mass than the expected full-length protein represent translation products derived from truncation frame shift or stop mutations in the analysed gene. As PTT is an RNA-based method, PTT is able to test several exons in one step instead of investigation each exon separately.

## Materials and Methods

### Patients

The clinical findings of all the patients suggested M.D., and the patients were referred to the Kennedy Center for molecular diagnosis of MD. The identified mutations in the 11 patients, confirming the diagnosis, are summarized in Table 1. Three normal controls were also included in the test.

### Cell culture conditions

The primary fibroblasts were cultured in a 1:1 mixture of RPMI 1640 with 20 mM Hepes buffer and Nutrient mixture F10 HAM media, supplemented with 7.5% Amnio Max C100 supplement, 4% foetal calf serum, glutamine, penicillin and streptomycin. The cells were incubated at 37 °C in sealed flasks.

### Preparation of RNA and cDNA

Total RNA was isolated from 10^4^–10^6^ cultured fibroblasts using the QIAgen RNeasy Mini Kit (QIAgen) and the RNA was eluted in 50 μl elution buffer. Single-stranded cDNA was synthesised with Superscript II RNAse H^−^ Reverse Transcriptase (Gibco BRL) using a mixture of random hexamer primers (Pharmacia) and 7.5 μl RNA solution in a total volume of 20 μl.

### Amplification of RT-PCR fragments followed by PTT

The cDNA amplifications of Menkes cDNAs were performed as nested PCR reactions. The PCR amplifications were done using 5 Units of Taq Plus Long (Stratagene) in High Salt buffer in a total volume of 40 μl containing 100 nM of each primer and 100 μM dNTP. 2 μl of cDNA was used as template in the first PCR reaction whereas 2 μl of the first PCR reaction was used as template in the second and final PCR reaction. The PCR primers used in the first PCR reaction were MNK-41U (ccataggatagagaaacc, -41-24 upstream the ATG start codon) and MNK-1962L (aagaccgtctccattgtct-tattt, corresponding to the sequence encoding amino acid 654–647), while MNKT7ATG-U (taatacgactcactatagggagaccaccatggatccaagtatgggt-gtgaat, corresponding to the sequence encoding amino acid 1–8 position) and MNK-1962L (atcta-agtgacttgctgaccgatcctt, corresponding to the sequence in exon 8, encoding amino acid 642–634) were used in the second nested PCR. The 5′ primer MNKT7ATG-U contains a T7 RNA-polymerase promoter sequence and a Kozak sequence in front of the ATG start codon. PCR amplifications were performed for 40 cycles (94 °C for 30s, 55 °C for 1 min and 72 °C for 3 min) followed by 7 min at 72 °C. The products were analysed on 2% agarose gel to verify amplification. For PTT, 4 μl of the amplified PCR product was translated at 30 °C for 90 min using the TnT^™^ T7-coupled reticulocyte lysate translation system (Promega, Madison, WI) in the presence of ^35^S methionine (Amersham Pharmacia) in a total reaction volume of 25 μl. Translation products were separated on a 10% SDS-PAGE gel under reducing conditions, followed by fixing (fix-buffer: 10% v/v acetic acid and 50% v/v ethanol) and incubation for 30 min in Amplify Amersham NAMP 100 (Amersham Pharmacia). The gel was dried before exposure to Kodak X-Amat AR film, XAR–5, at −80 °C for at least 24 h.

## Results

DNA from eleven patients with known mutations in the coding region from exon 2 to exon 7, corresponding to the encoding sequence c.1-c.1869, were chosen to verify the effect of the mutations and to demonstrate the efficiency of PTT. All mutations identified so far in this region, including those eleven, lead to a premature termination codon.

The effect of the truncation mutations present in the eleven MD patients were analysed by PTT. The cDNA sequence encoding the N-terminal part of ATP7A, corresponding to the segment from basepair 1 in exon 2 to basepair, 1927 in exon 8 (Genbank NM_000052, +1 corresponds to the A of the ATG translation initiation codon) was amplified by RT-PCR using RNA isolated from cultured fibroblasts as template. The mutations in the eleven tested patients were predicted to introduce a termination codon after 200, 221, 289, 304, 312, 518, 541, 546, 547, 593 or 624 amino acids respectively (Table 1). The PCR product amplified from the control RNA direct the synthesis of a protein containing 642 amino acids residues. The mutations were a mixture of single nucleotide mutations leading to termination codons, insertions or deletion of few basepair as well as a large deletion of two exons.

**Figure 1.**
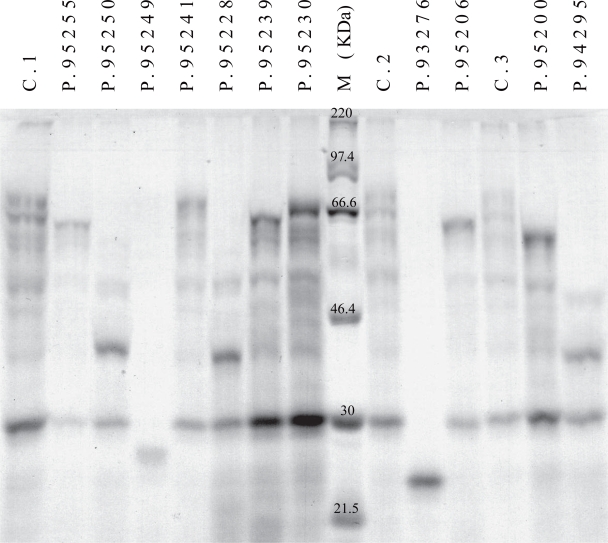
PTT of RT-PCR fragments spanning exon 2 to exon 7 of the ATP7A gene. M: Molecular-mass-marker proteins. C1, C2, C3: wild-type proteins obtained from three control samples. P: patients (patient number refers to Table 1).

The resulting protein products obtained after in vitro translation were analysed by SDS-PAGE (Fig. 1). While the size of the peptides in ten of the samples were clearly smaller than the size of the fully translated run-off product, corresponding to 642 amino acids, obtained in the control sample, the size of the peptide obtained in MNK95241 (Fig. 1) was barely detectable from the control product. However, the pattern was clearly different. While two bands were observed in all the control samples (C.1; C.2 and C.3) with an apparently molecular weight of 66 kDa and 60 kDa respectively, only one band at approximately 63 kDa could be observed in MNK95241. The mutation, a seven base pair deletion, in MNK95241 is located in exon 7 at nucleotide position c.1752–1758. Although, this deletion leads to a shift in the reading frame, formation of a premature termination codon was not obtained until further 114 nucleotides downstream, close to the position of the reverse primer used for PCR amplification. This mutation leads in total to an open reading frame encoding 624 amino acids, only 18 amino acids shorter than the wild-type fragment. A difference of only 18 amino acids is close to the limit of the resolving SDS-PAGE system. As a negative control, a sample containing water instead of a PCR product was investigated by PTT. This sample did not lead to any radioactive band after SDS-PAGE (not shown).

## Discussion

Comparison of the predicted length of the individual mutated protein products with the migration rate of the *in vitro* translated products revealed a perfect correlation (Fig. 1; Table 1). Interestingly, the mutation with the large deletion in sample, 95200, could have been detected by simple agarose gel electrophoresis of the RT-PCR products. As few percentages of all mutations in this region are large deletions or splice site mutations this quick procedure should be used as an initial screening.

False-negative results might derive from mutations near the extreme N or C-terminus of the protein product. If the mutation is located close to the reverse primer sequence there is a risk that the mutation will not be detected if the resulting premature termination codon is located too close to, or even beyond the sequence of the reverse primer. If the mutation is located in the forward primer-binding site the mutation will not be detected as long as a PCR product is produced, because the wild type primer sequence will be incorporated in the resulting PCR product.

In autosomal inherited disorders, where two alleles needs to be investigated, PTT can leads to false negative results because in some cases, only the normal mRNA is analysed due to nonsense mediated decay of the truncated transcripts (Kahmann et al. 2002). This problem have forced scientist to develop more sensitive non-radioactive PTT tests (Kahmann et al. 2002). As MD is a X-linked recessive disorder, the risk for false negative results as a result of allelic exclusion, can be left out of account.

In our experience PTT turned out to be a sensitive and effective method for detection of translation termination mutations, including nonsense mutations, deletions and insertions in the N-terminal part of the ATP7A gene.

In addition to the two products of about 66 kDa and 60 kDa a third product of approximately 30 kDa could be observed in the control sample. The reason for the presence of the 60 kDa and the 30 kDa product is unknown. As only one product at about 63 kDa is seen in sample MNK95241, it seems that the open reading frame in the immediate vicinity of c.1752 in the wild type provokes the formation of a 60 kDa truncated product, corresponding to a product terminated immediately after the sequence encoding copper binding site number six. The additional band of 30 kDa could also be observed in the major part of the other samples. The 30 kDa product was only absent in the samples MNK93276 and MNK95249, the only samples encoding a truncated products below 30 kDa. The 30 kDa product could represent a degradation product or more likely be the result of premature termination of the protein translation, taking place after approximately 270 amino acids leading to a protein consisting of the two N-terminal copper-binding sites. Further experiments might disclosure if truncated products composed of two respective six copper binding domains are produced in vivo and furthermore if they have any biological function.

While PTT appears to be an efficient and effective method to screen for point mutations in the region from exon 2 to exon 7 of the ATP7A gene, other methods needs to be used for the rest of the exons. For this we routinely use sequencing.

## Figures and Tables

**Table 1:** 

**Patient**	**Exon**	Nucleotide mutation*	**Mutation type**	**Consequence**
93276	3	c.601 C > T	nonsense	p.R201X
95249	3	c.609delA	deletion	Frameshift. Stop at codon 222
94295	4	c.869C > A	nonsense	pS290X
95228	4	c.796_798delTCAinsCC	deletion	Frameshift. Stop at codon 305
95250	4	c.893_897delCTTTA	deletion	Frameshift. Stop at codon 313
95200	6 and 7	c.1554-?_1869+?del	Partial gene deletion	Frameshift. Stop at codon 519
95206	6	c.1603_1606delGCTGinsTATACC	indels	Frameshift. Stop at codon 542
95255	6	c.1639C > T	nonsense	p.R547X
95239	6	c.1642G > T	nonsense	p.E548X
95230	7	c.1782C > G	nonsense	p.Y594X
95241	7	c.1752_1758del TAGTCTC	deletion	Frameshift. Stop at codon 625

* Nucleotide numbering of ATP7A cDNA is according to the ATG start codon. *ATP7A* reference sequence: NM_000052 with numbering starting with 1 at the A in the ATG codon.
